# Forces applied at the footrest during ergometer kayaking among female athletes at different competing levels – a pilot study

**DOI:** 10.1186/s13102-018-0113-5

**Published:** 2019-01-09

**Authors:** Å. B. Tornberg, P. Håkansson, I. Svensson, P. Wollmer

**Affiliations:** 1 Department of Health Sciences, Lund University, Baravägen 3, 221 85 Lund, Sweden; 2 Division of Solid Mechanics, Lund University, Box 118, 221 00 Lund, Sweden; 3 Centre of Biomechanics, Lund University, Lund, Sweden; 4 Department of Clinical Sciences, Malmö, Clinical Physiology and Nuclear medicine Unit, Lund University, 221 05 Malmö, Sweden; 5 Malmö Sports Academy, Malmö, Sweden

**Keywords:** Biomechanics, Maximal exercise test, Motion analysis

## Abstract

**Background:**

Power output and force development during exercise are thought to be important indices of performance in elite athletes. The aim of this preliminary study was to determine the forces applied at the footrest during ergometric kayaking in individual kayakers at different competitive levels.

**Methods:**

Three elite female kayakers participated voluntarily in the study. Oxygen consumption (VO_2_) and mean power were measured during paddling at three different work levels (15 W below onset of blood lactate accumulation (OBLA), at OBLA, 15 W above OBLA and all-out paddling) on a modified kayak ergometer. External force sensors were attached to the wires on right and left side connecting the paddle to the flywheel of the kayak ergometer. Individual footrests were built to enable measurements of pushing and pulling forces and to distinguish between the left and right foot.

**Result:**

The relative differences between the three athletes were similar for power, VO_2peak_ and forces at the paddle. There were, however, differences in the forces applied at the footrest, where the most accomplished paddler generated forces 3 to 26 times as high as the least accomplished paddler.

**Conclusion:**

The relative differences between the three athletes were similar for power, VO_2_ and forces at the paddle. There were, however, dramatic differences in the forces applied at the footrest.

## Background

In analysis of elite sport performance, ergometers are constructed to reflect the specific sport performance analysed [1]. The ergometers measure the work or power during the test [2] and sometimes also assess specific performance indices such as distance, time and speed. Few ergometers, however, provide information about force development during exercise. In kayaking, air braked kayak ergometers are often used for measurement of power output [1]. Previous testing on kayak ergometers [3] has been shown to reflect the physiological response during flat-water kayaking.

Kayaking engages large muscle groups to drive the kayak as fast as possible through the water. The kayaker has to overcome the drag force, which acts in the opposite direction to movement. Hydrodynamic (boat) and aerodynamic (boat and paddler) drag is generated as the kayak moves through the water [4]. The paddle in the water acts to transmit the net forces developed within the kayak by the paddler to provide forward propulsion. The forces developed at the paddle driving the kayak through the water have been thought to be produced mainly by upper body muscles [5–7].

There have only been few studies of the forces developed within the kayak during paddling. Aitken and Neal [8] found peak forces of 200 N in sub-elite kayak paddlers to be applied to the paddle during kayaking. In elite kayak paddlers Baker [9] measured peak forces of about 375 N in men and 290 N in women at the paddle. Since force development at the foot stretcher during the driving phase of the stroke has been shown to influence power output in rowing [10], similar conditions may apply to kayaking. Most elite kayakers apply some form of fixation of the feet to the footrests at least during competition. This allows both pushing and pulling forces to be applied to the footrest. We are not aware of any previous studies of the forces at the footrest and seat in kayaking, as also pointed out in a recent review article [4].

The aim of this preliminary study was to determine the forces applied at the footrest during ergometric kayaking in individual kayakers at different competitive levels – junior elite, national elite and international elite.

## Methods

### Subjects

Three female kayakers (age range 20–29 years, weight range 67.6–74.9 kg, height range 169–180 cm) volunteered to participate in the study. All three subjects are members of a national team one junior kayaker, one national elite kayaker and one international elite kayaker. The study was approved by the regional ethical review board at Lund, Sweden (ETIK 2007/72). All subjects have consented to have the results presented together with results from competitions [11].

### Study design

The study was performed in association with the tests carried out by these elite kayakers as part of the monitoring of their training. Two subjects were tested on one laboratory and the third in a second laboratory. All tests were supervised by the same member of the research group. The subjects performed the exercise test on a modified kayak ergometer to enable power, oxygen uptake (VO_2_) as well as forces in the paddle and foot-rests to be measured simultaneously during kayaking. The kayakers exercised at four work levels, 15 W below onset of blood lactate accumulation (OBLA), at OBLA, 15 W above OBLA and maximal all-out. The work level where OBLA was expected was known from previous testing in all subjects. Between each work level, there were a two minutes rest sitting on the kayak ergometer. The subjects were familiar with the testing procedure. The subjects were told not to eat or drink coffee two hours before the test session. They were also told not to perform any heavy exercise 48 h before the tests.

### Exercise capacity

The kayakers exercised for four minutes 15 W below OBLA, at OBLA and 15 W above OBLA. Two kayakers performed a two minutes maximal all-out test and one kayaker performed a four minutes all-out test. We had to adhere to the standard protocol for the three athletes, which accounts for this difference. A modified kayak-ergometer (Dansprint PRO, Dansprint Asp, Hvidovre, Denmark) was used. VO_2_ was measured breath-by-breath (Oxycon Mobile, Jeager, Hoechberg, Germany) in one subject and with mixing chamber (Oxycon PRO, Jeager, Hoechberg, Germany) in two subjects. Both systems were validated against measurements with Douglas bags and repeated measurements have been performed showing a coefficient of variance for VO_2_ of 3%. Calibration of the gas sensors was performed before each test with a certified gas mixture. Air flow was calibrated before each test using a calibration syringe. The use of different equipment is explained by testing in two laboratories as described above. The subjects were verbally encouraged to exercise as hard as possible during the all-out test. Blood lactate was measured with in one minute after completion of each work level. Capillary blood samples were taken and analyzed with a photospectrometer (Biosen, EKF Diagnostic, Magdeburg, Germany).

### Force and power analysis

A kayak ergometer (Dansprint PRO, Dansprint Asp, Hvidovre, Denmark) used during the exercise capacity testing, was equipped with external force sensors attached to the wires connecting both left and right side of the paddle driving the flywheel of the kayak ergometer. Individual footrests for the left and right foot were built to enable measurements of pushing and pulling forces. Individual adjustments of the footrests were possible in accordance with the original footrest design. Foot straps were used to maintain the position of the foot during kayaking. The force sensors were manufactured for this specific application. The sensors attached to the wires are based on small and light steel rings fitted with strain gauges, while the foot-rest sensors, also based on strain gauges, were integrated in the foot-rests. The strain gauges are coupled in full a Wheatstone bridge, to make the force sensors insensitive to factors such as temperature and resistance in the sensor wires. The sensors were calibrated by applying known linear forces in the same range as the measured forces. Data signals, to assess forces at the paddle and the feet, from the force sensors were collected through electric cables with LabWeiw 8.0 via the data acquisition system DAQPad-6015 and SC-2345 from National Instruments (Austin, Texas, USA) and data signals, to assess power, from the kayak ergometer were collected with the Dansprint Analyser 1.09 (Dansprint Asp, Hvidovre, Denmark).

### Data analysis

#### Exercise-capacity

VO_2_ at 15 W below OBLA, at OBLA and 15 W above OBLA was defined as the mean value recorded during the last two minutes of paddling at each work level. The VO_2peak_ was defined as the mean values recorded during the last minute of the all-out test. Power was the mean value recorded on the kayak ergometer at each work level.

#### Forces

Forces at the paddle and footrest were recorded over two or four minutes depending on the duration of the maximal all-out test. The signal from unloaded sensors was taken as zero. A frequency distribution of force over time was generated and the 95th percentile of force was taken as peak force. This procedure was used in order to reduce the impact of noise in the measurement. The analysis was performed using LabWeiw 8.0 and Matlab R2006a.

## Results

**Fig. 1:**
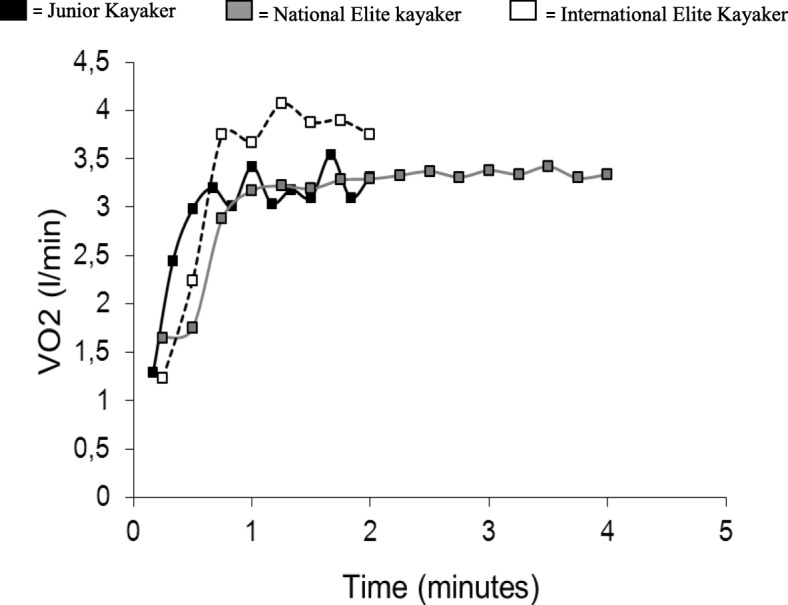
Oxygen consumption (l/ min) during the maximal all-out tests

**Fig. 2:**
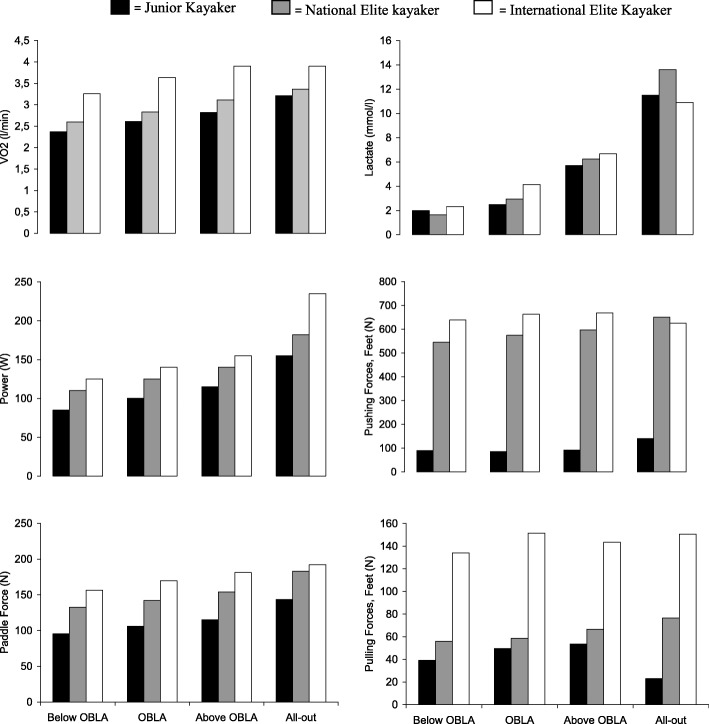
VO_2_, lactate, power, paddle force, pushing forces at the feet and pulling forces at the feet during ergometric paddling below OBLA, at OBLA, above OBLA and at all-out. Force values are presented as mean values of left and right side measurements

VO_2_ during the all-out test is shown in Fig. 1. All three subjects reached a plateau in VO_2_, though not as succinct in the junior kayaker, and respiratory exchange ratio exceeded 1.1 during the all-out test. The results of measurements during exercise are presented in Fig. 2. The subjects are sorted by increasing power during the test. As could be expected, the rank order was the same for power, VO_2peak_ and competition time, both at 500 and 1000 m (Table 1). The relative differences between the three athletes were similar for power (At All-out 155; 182; 235 W), VO_2peak_ (at All-out 3.2; 3.3; 3.9 L/minute) and forces at the paddle (At All-out144; 183; 192 N). There were, however, dramatic differences in the forces applied at the footrest (At All-out pull 23; 77; 150 N), where the most accomplished paddler generated forces 3 to 26 times as high as the least accomplished paddler (Table 1).

**Table 1:** VO_2_, lactate, power, competition time, paddle forces, pushing forces at the feet and pulling forces at the feet during ergometric paddling at all-out

	All-out (min)	Power (W)	VO_2peak_ (L min^−1^)	Paddle		Foot left		Foot right		Lactate	Competition time	
				(N)		(N)		(N)		(mmol l^−1^)	(min.sec)	
				Left	Right	Push	Pull	Push	Pull		500 m	1000 m
Junior Kayaker	2	155	3.21	145	142	175	7	106	39	11.5	2.13	4.37
National Elite Kayaker	4	182	3.36	179	187	716	75	585	78	13.6	2.00	4.19
International Elite Kayaker	2	235	3.9	187	197	584	186	667	115	10.9	1.54	3.57

## Discussion

The main finding of this study was that the kayakers at different competitive levels in this study generate considerably different forces on the footrest during paddling on a kayak ergometer.

Aitken and Neal [8] reported peak forces (right paddle 213.5 ± 9.6 N, left paddle 200.6 ± 7.9 N) measured with strain gauges on the paddle shaft during 500 m on-water kayak paddling in sub-elite kayak paddlers. Baker [9] has reported higher peak forces (men 1000 m 375 N, women 500 m 290 N) during on-water measurements in kayak paddlers from the Australian national team. Our findings are compatible with the previous studies, despite the fact that we had only three female subjects and that the relationship between force measurements on water and in a kayak ergometer is unknown.

We have measured forces at the footrest during kayaking. Forces at the footrest have previously been measured during rowing. Caplan and Gardner [12] thus found that mean power increased with pulling forces of the footrest. Pushing forces at the footrest during rowing have been proposed to relate proportionally to forces at the oar blade [2, 13]. In rowing the oars are attached to the boat [14]. This is mechanically different from kayaking, where there is no attachment between the paddle and the boat. In rowing, the forces from the oars act rather symmetrically on the boat and rower and the boat is pushed forward through the feet. In kayaking, the kayakers themselves have to transfer the force from the paddle to the boat via the body [6]. Since the force in the paddle blade, that drives the kayak forward, acts a distance away from the centre line of the kayak, every stroke will tend to turn the craft. This is partly compensated for in the boat design. The moment may also need to be balanced by sideway forces in the seat being stabilised by a pulling force in the foot at the opposite side from the active side. A high moment from a high power, could then be balanced from pulling force in one foot.

**Fig. 3:**
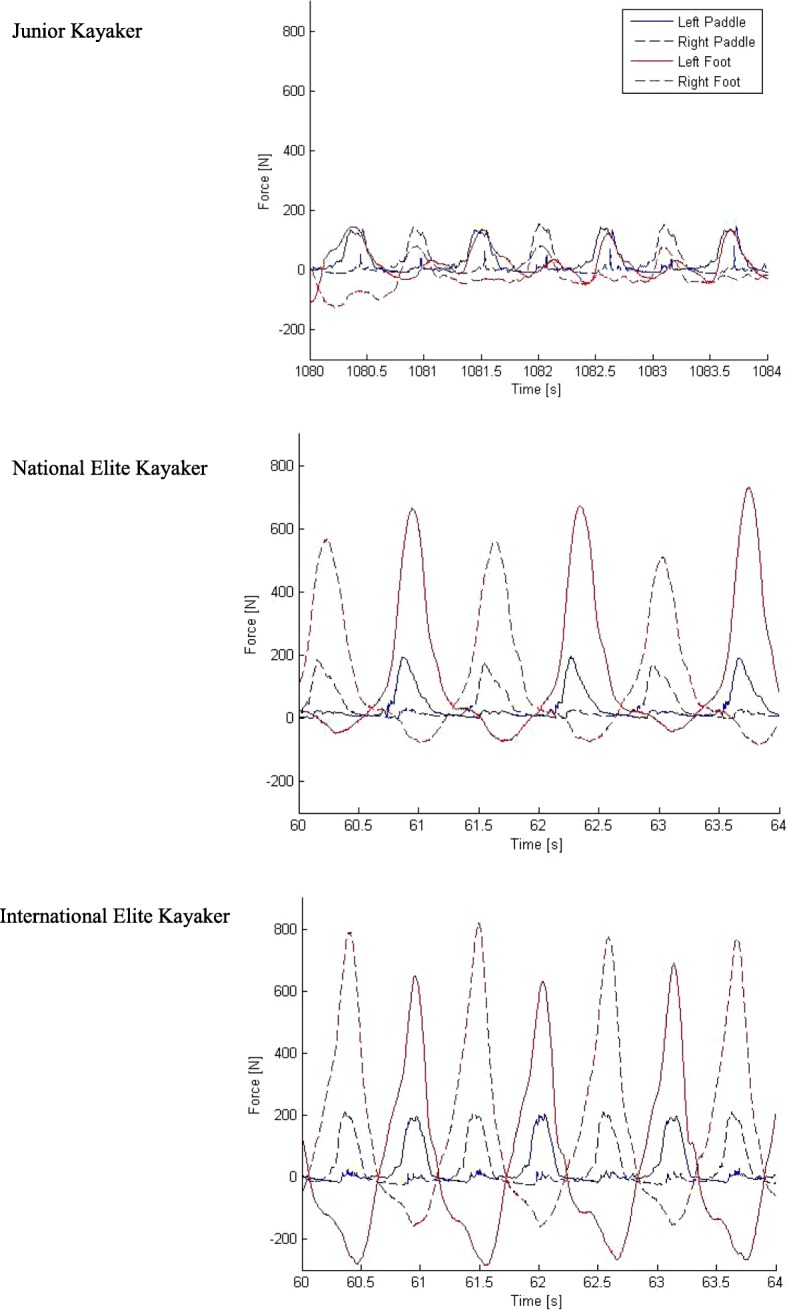
Paddle force, pushing forces at the feet and pulling forces at the feet during ergometric paddling during 4 s at All-out

Timing between upper body and lower body seems to be important during ergometric kayaking by looking at the force curves (Fig. 3) from the three athletes. Even further seems the coordination between the pushing and pulling movement also to be important. One could speculate that the timing of the moment is important to be able to produce high power. A paddle stroke involves rotation of the torso from the shoulders to the pelvis [4]. Rotation of the pelvis is likely to involve leg muscles and application of forces on the footrest may help stabilising the torso during the movement.

Earlier it has been suggested that high aerobic and anaerobic capacity are the most important factors distinguish between elite kayakers [5, 7]. Our findings suggest that timing and biomechanical factors as forces at the feet have greater impact on performance (Fig. 2).

Our preliminary results show that while the relative differences in power, VO_2_ and forces at the paddle were similar in the three athletes, there were very large differences in forces at the foot rest. Technical aspects of paddling, e.g. stroke profile and the direction of force at the contact between paddle and water may conceivably be related to generation of forces at the foot-rest. There are several limitations of this study. The low number of subjects is certainly one. We could only perform the study in association with routine testing during the competition season, which meant that slightly different protocols and different equipment had to be used. The results would however, be the same if we analysed the forces after two minutes in the kayaker who performed four minutes all-out tests. The equipment for measurement of VO_2_ had been validated in the same laboratory (Elite Sports Centre Bosön, Lidingö, Sweden), and we are confident that the two units give comparable results. Further studies of the forces at the footrest during kayaking and their relation to power and competition time are needed to confirm our findings. If such relations exist, specific training of the co-ordination between paddle stroke and forces applied at the footrest may help improving performance. Studies of the relation between power and forces with and without fixation of the feet may provide an insight into the importance of pulling forces at the footrest. Forces at the footrest should also be studied during paddling on water, as this may differ from ergometer paddling. More comprehensive measurements of forces and movements are needed to provide a full picture of the biomechanics of kayaking.

## Conclusion

Important indices of elite kayaking seem to be forces applied at the footrest as the kayaker performing at the highest level produced the largest forces. Timing and coordination of forces in the paddle and footrest, seems to be important for high kayaking performance. Suggesting that forces at the footrest could be an important indices to assess during training evaluation.

## Abbreviations

OBLA: Onset of blood lactate accumulation
VO_2_: Oxygen uptake
